# Male Nurses.—I

**Published:** 1890-10-18

**Authors:** 


### Passing Topics.

### MALE NURSES.—I.

The annual report of the Hamilton Association for providing trained male nurses is not wanting in interest, while the work it represents deserves more attention than it commonly obtains. The Hamilton Association, limited though its operations are, is virtually the only organization concerned with the training of men as nurses, and it appears little short of lamentable that a provision which common sense and the exigencies of actual practice alike show to be needful is left unsupplied for want of fitting opportunities to manipulate the raw material.

We believe we are right when we say that the managers of the Hamilton Association have made many and vain endeavours to obtain facilities for training young men at the metropolitan general hospitals, and that at the present time the only institution which opens its doors to men is special—the National Hospital for the Paralysed and Epileptic—an institution which, by reason of the nature of its work, stands at all times in need of the services of a certain number of male nurses.

We readily recognise the difficulty of training men and women together, but it may be fairly asked why, when the usefulness of male nurses in particular cases is generally admitted, a small proportion of wards in general hospitals cannot be put into the charge of men? The Association has very wisely deprecated any idea of interfering with the beneficent operations of women; but authorities are agreed that in certain cases the services of male nurses would not only be desirable on the score of the greater strength a man could bring to bear, but on the ground of propriety.

It is all very well to use fine expressions in reference to the work of a nurse, and to seek to hide the nature of duties which will not bear specific mention under rhetorical phrases. We yield to none in our admiration for the devoted labours of women in this field; but there are duties in regard to men patients the performance of which it is a positive offence to demand at a woman's hands. That this is not to overstate the case is made manifest by a perusal of the evidence of no less a personage than the president of the Royal College of Physicians, recently given before the Lords' Committee. Said Sir Andrew Clark, after paying a cordial tribute to the nurses, "at the same time there are cases occurring in a large general hospital where I think it indecent to have women as nurses; cases of delirium tremens, and a number of cases of that sort, in which I think male nurses ought to be employed exclusively."

Imagine a young and refined woman set to watch a case of this description for hours together—to witness scenes calculated to shock the most robust, and to listen to language foul and blasphemous beyond conception. Yet, as custom now is, even when fear of physical violence compels the assistance of a male attendant, the woman nurse is expected to remain "in charge" of the patient.

We have no hesitation about echoing the sentiment of many who have knowledge of this subject—that if only fathers, mothers, and brothers knew more of the duties their daughters and sisters are called too often upon to perform in their everyday experience as hospital nurses, there is no honest occupation they would not sooner see them take up with. That there are objections to the employment of male nurses which may be said to be inherent to the employment of men in any purely domestic duties may be admitted. Men do not always readily adapt themselves to the work of a sick-room. They want more liberty than women; they must have opportunities to smoke their pipes, and they look for higher wages. All this is undeniable, but it would be trifling with a serious subject to consider such difficulties insurmountable. Agricola.

